# Cows fed hydroponic fodder and conventional diet: effects on milk quality

**DOI:** 10.5194/aab-62-517-2019

**Published:** 2019-08-15

**Authors:** Alan Agius, Grazia Pastorelli, Everaldo Attard

**Affiliations:** 1 University of Malta, Division of Rural Sciences and Food Systems, Institute of Earth Systems Msida, MSD2080, Malta; 2 University of Milano, Department of Veterinary Medicine, Via Celoria 10, Milan, Italy

## Abstract

The technology of green fodder production is especially important in arid and semiarid regions. Hydroponics improves on average the amount of crops in the same space, as traditional soil-based farming and can reduce water consumption compared to traditional farming methods. Limited research has been carried out on the use of hydroponic fodder and milk quality.

A comparative study of traditional (Malta farm) and hydroponic fodder (Gozo farm) was conducted in Malta with 20 cows of the Holstein–Friesian breed from two farms. Individual and bulk-tank milk samples were collected once a week for a period of 1 month in order to evaluate physical (pH, conductivity, density, freezing point) and chemical (fat, protein, ash, lactose, solid nonfat) parameters as well as mineral (Zn, Cu, Pb, Ba) content. Milk proximate and physical data were processed by analysis of variance (ANOVA) for repeated measures and an ANOVA procedure with farm and time as effects for minerals.

The results indicated differences in fat content and pH, showing higher values (P<0.05) in milk samples of cows fed with the hydroponic rather than the traditional fodder; a significant time effect (P<0.001) was found in all qualitative analyses except for lactose and salts. Minerals were in the range as reported elsewhere; Cu and Pb content was significantly higher (P<0.001) in the Gozo farm than the one in Malta, whereas Zn content showed higher values in Malta (P<0.001) than Gozo. Although the proximate results were similar for both farms, except for the higher fat content for the Gozo farm, principal component analysis (PCA) revealed that milk quality for the Gozo farm was superior to that of the Malta farm. However, further studies are needed to determine the effects of different hydroponic fodder using a large herd size.

## 1 Introduction

The Maltese islands are situated at the center of the Mediterranean Sea, with two major inhabited islands: Malta and Gozo. Their surface areas are approximately 300 and 100 km${}^{2}$, respectively. There are four dairy cattle breeds, which are Jersey, Brown Swiss, Estonian Red Cattle, and Holstein–Friesian. The latter breed is the most common one on the islands. In 2014, there were 6502 heads in 85 dairy cow farms (NSO, 2016).

Milk composition is economically important to milk producers and processors, and it is nutritionally important to consumers. It has been known for years that variations in milk composition occur. However, the strong selection of cow breeds, particularly over the last 50 years, has reduced this type of variability considerably (Huppertz and Kelly, 2009). The composition of milk is made up of fats (on average making up 4 % of the milk), proteins (3.2 %), other “solids” (5.3 %), and water (87.5 %) (European Commission, 2006). Factors that affect milk composition include breed, genetic variation within breed, health, environment, management practices, and diet. Among the different dietary regimens worldwide, hydroponic fodder production is now widely accepted. There are some arguments about sprouting grains for convenience in green forage production with hydroponic systems to compensate for the feed resources for animals (Hassan et al., 2016). Hydroponic green fodder is produced from forage grains, having a high germination rate and growing over a short period of time in a special chamber that provides the appropriate growing conditions (Fazaeli et al., 2011). The development of this planting system has enabled the production of fresh forage from oats, barley, wheat, and other grains (Rodriguez-Muela et al., 2004).

In Malta, out of 11 428.5 ha utilized for agriculture, 5552.8 ha are used for the production of fodder crops (NSO, 2012). The fodder is either allowed to dry in situ and then harvested in bales or else harvested and silaged. Additional feed is imported and in some instances transformed into concentrates by local feed mills. Unfortunately, cows do not acquire the benefits of fresh pasture. The hydroponic fodder system is an ideal solution in places where there is limited land area for the growing of fodder or where pasture grazing is limited or nonexistent. It is also sustainable as it occupies a small land area, thus making it ideal for limited areas such as the farms in Malta. This system provides a considerable amount of fresh fodder with a high nutritional value every day at a low cost. Hence, this research aims to evaluate milk quality in terms of physicochemical characteristics and assess the content of Cd, Pb, Cu, and Zn in milk coming from dairy farms in which different feed regimens (traditional and hydroponic) are given.

## 2 Material and methods

### 2.1 Animals and experimental design

The experiment was conducted on 20 cows of the Holstein–Friesian (HF) breed from two farms, one from Malta (ML) (N=10) and the second one from Gozo (GZ) (N=10). The two farms had similar management conditions. Cows were selected according to the following conditions: milk production (20±5 kg d${}^{-1}$) and parity equal to 2–3. The cows were kept in traditional tied-up barns. The composition of the two diets is reported in Table 1.

Recently, particularly in GZ, farmers are feeding fresh hydroponic grass every day. This is mixed with hay (alfalfa), local straw, silage, and concentrates, while others are feeding hay, local straw, and concentrates to their cows. Both farms feed nearly the same concentrates, but with different rations and ingredients, using the total mixed ration (TMR) system. Feed was offered to achieve 5 % refusal on the basis of the feeding practice followed on both farms. The cows were milked two times per day at 05:30 and 15:00 and at 07:00 and 16:00 (GMT+2 h), respectively, in GZ and ML farms. The TMR was fed daily at approximately 11:00. Water is available ad libitum. The experiment was conducted in such a way that the usual daily farm activities were not disrupted, but it was ensured that all housing and animal care conditions conformed to the standards recommended by the Guide for the Care and Use of Laboratory Animals, Directive 2010/63/EU and Directive 1998/58/EU.

**Table 1:** Ingredients and chemical composition (% of DM) of Malta (MT) and Gozo (GZ) diets.

	Farm	
Composition	MT	GZ
Ingredient		
Hay${}^{1}$	21.37	–
Local straw ${}^{2}$	5.79	26.28
Ground maize${}^{3}$	4.83	10.14
Extrolin${}^{4}$	2.71	3.16
Normal dairy pellets${}^{5}$	45.60	5.36
Special dairy pellets${}^{6}$	19.70	23.00
Corn silage${}^{7}$	–	9.15
Alfalfa hay${}^{8}$	–	15.33
Hydroponic${}^{9}$	–	7.58
Calculated chemical composition${}^{10}$		
Dry matter, %	88.16	59.10
Crude protein	13.33	13.48
Fat content	3.75	3.76
Ash	8.70	9.07
NDF	49.06	46.16
ADF	19.00	21.79

${}^{1}$ Hay had a DM of 88.72 % and chemical composition (DM basis) of 1.92 % EE, 12.62 % CP, 35.55 % ADF, 57.41 % NDF, 10.82 % ash. ${}^{2}$ Straw had a DM of 86.18 % and chemical composition (DM basis) of 1.33 % EE, 12.85 CP, 36.83 % ADF, 61.3 % NDF, 12.64 % ash. ${}^{3}$ Ground maize had a DM of 86.29 % and chemical composition (DM basis) of 4.68 % EE, 6.18 % CP, 48.23 % NDF, 1.62 %, 1.62 % ash. ${}^{4}$ Extrolin had a DM of 96.82 % and chemical composition (DM basis) of 20.57 % EE, 29.44 % CP, 1.42 % ADF, 7.24 % NDF. ${}^{5}$ Normal dairy pellets supplied by Kooperattiva Produttori Tal Halib Ltd. (Malta), containing maize, barley wheat pollards, soybean meal, and alfalfa (closed formula). Additives provided (per kg): Zn 70 mg; Mn 50 mg; Fe 20 mg; Cu 20 mg; I 1 mg; Se 0.4 mg; Co 0.25 mg; 10000 IU vit. A, 2000 IU vit. D3. ${}^{6}$ Special dairy pellets supplied by Kooperattiva Produttori Tal Halib Ltd. (Malta), containing soybean meal, maize, protected fats, wheat pollards, and barley (closed formula). Additives provided (per kg): Zn 140 mg; Mn 90 mg; Fe 31 mg; Cu 20 mg; I 4 mg; Co 0.7 mg; Se 0.5 mg, 36000 vit. A IU, 3600 IU vit. D3, urea, 12 mg. ${}^{7}$ Corn silage had a DM of 35 % and chemical composition (DM basis) of 7.4 % EE, 8.3 % CP, 33.90 % ADF, 55.3 % NDF, 10.8 % ash. ${}^{8}$ Alfalfa hay had a DM of 88 % and chemical composition (DM basis) of 1.28 % EE, 18.00 % CP, 34.55 % ADF, 44.00 % NDF, 12.00 % ash. ${}^{9}$ Hydroponic had a DM of 15.83 % and chemical composition (DM basis) of 0.51 % EE, 12.00 % CP, 5.76 % ADF, 12.73 % NDF, 2.17 % ash. ${}^{10}$ Values were obtained from a chemical analysis of feed (DM: dry matter; EE: ethyl ether fat extract; CP: crude protein, ADF: acid detergent fiber; NDF: neutral detergent fiber).

## 3 Production of hydroponic barley fodder (Gozo farm)

Hydroponic fodder barley was produced in a hydroponics chamber measuring about 4 m × 7 m × 2.5 m, with a daily production potential of 500 kg fresh hydroponic barley fodder and equipped with an automatic water-sprayer irrigation system. This system consists of a well-insulated room with climate control, extractors, and automatic sprinklers. The room consists of shelves where the plastic growing trays are placed and are watered with sprinklers, which work automatically without any wastage. Every day the trays are shifted from one row to another to complement the 7 d cycle from germination to harvest.

### 3.1 Fodder samples and analytical methods

Weekly samples of individual ingredients were collected and subjected to chemical analysis. Samples were analyzed in triplicate for chemical composition using an Infrastar 1400RTW near-infrared spectrophotometer (Unity Scientific, Brookfield, USA), and values for crude protein, ethyl ether extract (fat), ash content, neutral detergent fiber (NDF), and acid detergent fiber (ADF) were used to calculate the chemical composition of the TMR (Table 1).

### 3.2 Milk sample preparation

Individual and bulk-tank milk samples were taken once a week for a period of 1 month, from mid-November to mid-December 2013. A total of 88 samples were collected. The selected sampling day was fixed for both farms throughout the experimental period. The samples were taken in the morning and were analyzed in the afternoon. The individual milk samples were taken from a container connected to the pipeline system as soon as the cow stopped milking. All individual samples were labeled with the same ear tag number of the cow and were kept in a refrigerator (4 ${}^{\circ }$C); before analysis, the temperature of the samples was acclimatized at room temperature. The samples were tested for proximate parameters (physicochemical characteristics and metals). A bulk-tank milk sample was collected in order to evaluate the overall milk quality of the herd. The milk samples were stored in sterile tubes. The samples from GZ were sent to MT in a cooler (4 ${}^{\circ }$C) to preserve the properties of the milk for the analysis.

### 3.3 Physicochemical analysis

A MasterPro LM2 (Milkotester Ltd., Belovo, Bulgaria) was used to measure protein, fat, freezing point, density, solid nonfat, lactose, salts, added water, conductivity, and pH in milk samples. The principle of such instruments is the measurement of turbidity, or light scattering, caused by fat globules in milk. All samples were analyzed in triplicate.

### 3.4 Mineral concentration

The concentration of Zn, Cu, Pb, and Ba in the bulk and individual milk samples was determined by a microwave plasma–atomic emission spectrometer (MP-AES; Agilent 4100, Agilent Technologies, Santa Clara, USA) after mineralization. Briefly, milk samples (2 mL) were treated with 5 % ${\mathrm{HNO}}_{3}$ and heated on a hot plate for approximately 75 min. The samples were ashed at a temperature of 500 ${}^{\circ }$C for 4 h and made up to a 50 mL volume by dissolving the ashes in 5 mL of 5 % nitric acid topped with deionized water (Spiteri and Attard, 2017).

Each metal was calibrated against a known set of standardized concentrations at the optimal wavelengths (nm). The instrument and metal parameters are listed in Tables 2 and 3.

**Table 2:** MP-AES configuration and operating conditions.

Pump speed (rpm)	15
Sample introduction	Manual
Stabilization time (s)	15
Uptake time (s)	15 (using fast pump)
Nebulizer	Agilent OneNeb pneumatic concentric nebulizer
Spray chamber	Double-pass glass cyclonic spray chamber for Agilent MP-AES
Elements, wavelengths (nm)	Zn (213.857), Cu (324.754), Pb (405.781), Ba (455.403)

**Table 3:** Figures of merit from the MP-AES method representing the wavelength of detection, correlation coefficient ($R^{2}$), limits of detection (LOD), and limits of quantitation (LOQ) of metals under study.

Element	Wavelength	$R^{2}$	LOD	LOQ
	(nm)		(ppm)	(ppm)
Zn	213.857	0.9964	0.00819	0.02480
Cu	324.754	0.9996	0.00228	0.00690
Pb	405.781	0.9991	0.01743	0.05282
Ba	455.403	0.9774	0.00004	0.00012

### 3.5 Statistical analysis

Milk proximate data were analyzed using a repeated measurement analysis procedure with farm and time as the main effects. Farm, time, and time–farm combinations were included in the model. For metal analysis, a factorial analysis of variance (ANOVA) model with second-order interaction was applied, considering farm and time as fixed factors; the Student–Newman–Keuls (SNK) test for multiple comparisons was applied. The effect of replicate samples was tested separately, was not significant for any of the examined parameters, and was omitted from the model. Principal component analysis and Pearson correlations were conducted on all samples using XLSTAT v.2014.4.04 (http://www.xlstat.com, Addinsoft; last access: 8 August 2019) to determine any clustering for the proximate values of milk quality. Treatment effects were deemed significant at P<0.05, and a trend was noted when P<0.10.

## 4 Results and discussion

Recently, interest has been renewed in the utilization of fresh forage for dairy cows. Where dairying relies on the sole use of a TMR for feeding dairy cows, this interest in fresh forage may be justified when feed costs increase along with greater volatility in the price of conventional feeds (Menoza et al., 2016). The hydroponic fodder system is an ideal solution in Malta, providing a large amount of fresh fodder every day with minimal nutritional input, which efficiently encourages rapid growth.

In fact, it is ideal for areas where it is not possible to grow fresh fodder in a field over a very short period of time all year round. Studies have shown that this system may help the dairy cattle by increasing the fat percentage and health benefits of the herd (Naik et al., 2015).

This study was carried out to investigate whether the consumption of hydroponic fodder affects milk quality. To determine whether the sampled cows represented the herd, the bulk tank was sampled alongside the individual cows. The results of the proximate analysis of the average milk values of the 10 cows and the bulk tank (Table 4) did not show significant variations (P>0.05). Slight variations may have been due to the different handling procedures adopted for the individual samples vis-à-vis the bulk samples. Whereas the individual cow samples were obtained directly from the cow and then preserved at refrigeration temperatures, the bulk-tank samples were obtained from the herd's milk collected in a chiller.

The hydroponic fodder system produces fresh daily forage with a high nutritional value. Barley is placed in growing trays without any type of medium and allowed to grow over a period of 7 d. Germination starts within 24 h with a high germination rate of 85 % or better depending on how efficiently the temperature and light are maintained inside the system. Artificial light is used in this system to maximize growth, and special wavelength lights are strategically positioned to illuminate the seed beds for maximum growth. At the end of each cycle, hydroponic grass yields a constant layer of sproutings and densely packed roots. In 7 d the fodder crops, typically barley, reach a height of around 15–20 cm. The dairy cows consume the entire material without any waste because it is all edible and fresh. Hydroponic fodder requires no additional nutrients because it uses the resources and energy from the seed itself. This system requires a regular single-phase power supply, a potable water supply, and a low operating cost with minimum labor requirements. It only takes around 1 h of work per day to clean the trays, reseed, and harvest. With this system fresh grass is harvested daily all year round regardless of the weather. Setting up this system is more sustainable than importing hay from other countries as the cost of transport is expensive and the feed quality is inferior. On the other hand, hydroponic fodder is cheaper, resulting in improved milk quality (Carrillo et al., 2013). The cost of a bale of hay is much higher than cultivating fresh fodder each day. Some studies have showed controversial results with this system, and the relationship between the hydroponic system and milk quality is not always positive. Saidi and Omar (2015) observed no difference in the composition of milk from ewes fed with hydroponic barley. In the present work, carried out on cows, the average milk fat content in GZ was 4 % and that in MT for the 4-week period was 3.5 % (Table 4). Analysis of variance revealed the following results: GZ milk had a higher fat content and a higher pH. Fat is a main component in milk and has important nutritional and technological properties. About 98 % of milk fat is represented in the form of triglycerides, which are essentially esters of glycerol and fatty acid. The properties of milk fat are to a large extent determined by the fatty acid composition (Larsen et al., 2014).

**Table 4:** Effect of feeding system on milk chemical composition (average of individual cows with bulk-tank values in brackets).

	Farm		P value		
	Malta	Gozo	Time (T)	Farm (F)	T×F
Fat, %					
Week I	3.70 (3.60)	4.54 (4.00)			
Week II	3.07 (3.10)	4.05 (4.00)			
Week III	3.36 (3.10)	4.54 (4.50)			
Week IV	3.00 (3.30)	3.58 (4.20)	0.000	0.01	ns
Protein, %					
Week I	2.63 (2.60)	2.89 (2.70)			
Week II	2.67 (2.70)	2.82( 2.70)			
Week III	2.63 (2.60)	2.70 (2.70)			
Week IV	2.68 (2.60)	2.63 (2.70)	0.000	ns	0.000
SNF, %					
Week I	7.33 (7.26)	7.99 (7.59)			
Week II	7.44 (7.37)	7.76 (7.55)			
Week III	7.29 (7.10)	7.49 (7.38)			
Week IV	7.46 (7.10)	7.34 (7.38)	0.000	ns	0.00
Density, kg m${}^{-3}$					
Week I	26.2 (25.9)	29.8 (26.9)			
Week II	26.9 (26.7)	27.6 (26.8)			
Week III	26.2 (25.6)	26.2 (28.5)			
Week IV	27.1 (25.5)	26.2 (26.0)	0.000	ns	0.00
Lactose, %					
Week I	3.98 (3.90)	3.93 (4.10)			
Week II	4.04 (4.00)	4.20 (4.10)			
Week III	3.95 (3.90)	4.04 (4.00)			
week IV	4.06 (3.80)	3.98 (4.00)	ns	ns	ns
Ash, %					
Week I	0.56 (0.50)	0.56 (0.60)			
Week II	0.57 (0.60)	0.59 (0.60)			
Week III	0.55 (0.50)	0.57 (0.60)			
Week IV	0.58 (0.50)	0.57 (0.60)	ns	ns	ns
pH					
Week I	6.12 (6.20)	6.50 (6.60)			
Week II	6.38 (6.30)	6.59 (6.50)			
Week III	6.54 (6.50)	6.60 (6.60)			
Week IV	6.58 (6.50)	6.62 (6.60)	0.00	0.00	0.00
Conductivity, mS cm${}^{-1}$					
Week I	4.65 (4.60)	4.64 (4.70)			
Week II	4.70 (4.70)	4.69 (4.70)			
Week III	4.67 (4.60)	4.73 (4.70)			
Week IV	4.67 (4.60)	4.68 (4.70)	0.004	ns	0.092

Ns: not significant

**Table 5:** Milk mineral content in two different farms during the observed period. Bold values represent a correlation between parameters at p<0.05.

Variables	SNF	Density	Fr. point	Protein	Lactose	Salts	Added water	Temp.	pH	Cond.
Fat	**0.560**	0.420	0.097	**0.553**	0.234	0.283	-**0.540**	-0.167	**0.525**	0.369
SNF		**0.981**	-0.409	**0.994**	**0.581**	**0.599**	-**0.882**	-0.093	0.283	**0.774**
Density			-**0.446**	**0.976**	**0.578**	**0.588**	-**0.844**	-0.086	0.272	**0.766**
Fr. point				-**0.442**	-0.200	-0.246	0.302	0.118	0.213	-**0.558**
Protein					**0.578**	**0.582**	-**0.856**	-0.081	0.279	**0.764**
Lactose						**0.949**	-**0.579**	-0.304	0.009	0.398
Salts							-**0.689**	-0.390	0.094	**0.457**
Added water								0.287	-0.222	-**0.762**
Temp.									-0.174	-0.288
pH										0.131

The fatty acids in milk originate from different sources: de novo synthesis in the mammary gland, body fat reserve, and fatty acid produced from bacteria in the rumen as recently reviewed (Hanuš, et al., 2018). Young fresh fodder contains more fatty acids than other forages, which are harvested when they mature (Elgersma et al., 2015), and contains fatty acids that improve the quality of the milk. It is hypothesized that the transcription of enzymes involved in milk fat synthesis is affected by grazing fresh forage containing large concentrations of polyunsaturated fatty acids (PUFAs) (Wiking et al., 2010). The high fat content found in the present paper is likely due to a major PUFA content in feed (such as silage and hydroponic fodder) not consumed in the MT farm. The milk protein content of cows in both farms was quite similar (P>0.05). The GZ farm had a range from 2.8 % to 3.2 %, while the one in MT was 2.6 % -2.9 %. Density in milk is used to estimate the solid content in milk. The milk density did not differ between the farms, showing a value of 27.5 kg m${}^{-3}$ in GZ and 26.6 kg m${}^{-3}$ MT (P=0.179). Analysis of the freezing point in milk is an important indicator of milk quality (Zagorska and Ciprovica, 2013; Hanuš et al., 2013). The freezing point in milk is mainly determined to detect adulteration of milk with added water. Milk analysis may be used as a tool to determine whether the farmer added water to the milk to increase volume for a better but fraudulent return. This parameter did not vary between farms, having values (data not shown) within the normal range (-0.555 ${}^{\circ }$C). Conductivity is a typical test used to detect clinical and subclinical mastitis soon after infection. Electrical conductivity is a measure of the resistance of milk to an electric current, and conductivity is inversely related to electrical resistance. During infection with mastitis, the milk concentration of lactose and $K^{+}$ are decreased, and concentrations of ${\mathrm{Na}}^{+}$ and ${\mathrm{Cl}}^{-}$ are increased because of increased blood capillary permeability, the destruction of tight junctions, and the destruction of the active ion pump system. All conductivity results for each cow were analyzed to verify if the cows had a problem with mastitis or not. Cows from both farms did not exhibit any problems with mastitis, as all values were within the ranges quoted elsewhere. The average for both farms was 4.68 mS cm${}^{-1}$. Lactose was within the ranges quoted elsewhere (Scrimshaw and Murray, 1998) and salt contents were lower than those reported by Fox et al. (2015). Although the salts of milk are quantitatively minor constituents (Gaucheron, 2005), they are of major significance to its technological properties (Bijl et al., 2013). In the present study, no difference was found between the two farms. As quoted elsewhere, some differences in the milk salt concentration may occur during the change in season or during mastitis, but this was not the case with the present study. Moreover, in this study, the salt content was specifically studied from a mineral point of view as discussed later. Factor analysis using principal components was used to identify latent traits within the data. Pearson correlation (Table 5) revealed that there were several correlations between parameters.

**Figure 1:**
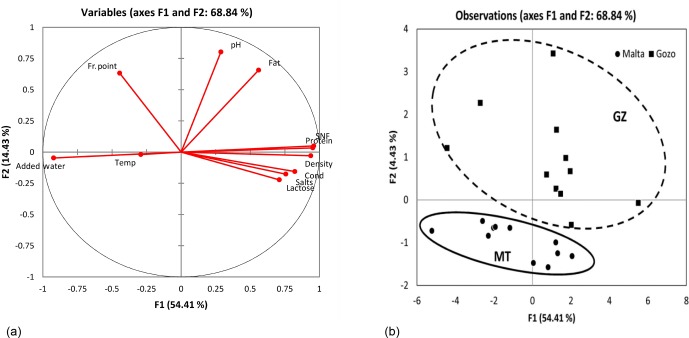
**(a)** The factor loading plot demonstrating the different groups of variables; **(b)** the factor scores of the two latent factors.

Solid nonfat (SNF) correlated positively with density, protein, and conductivity but negatively with added water (r=0.981, 0.994, 0.774, and -0.882 respectively). Protein correlated positively with SNF, density, and conductivity but negatively with added water (r=0.994, 0.976, 0.764, and -0.856). Added water correlated negatively with all parameters except freezing point. Conductivity correlated positively with SNF, density, and protein but negatively with added water (r=0.774, 0.766, 0.764, and -0.762, respectively). Two latent factors had an eigenvalue greater than 1, which together explained 68.84 % of the total variance (Fig. 1a). The factor loadings demonstrated the different groups of variables. For the first factor, the factor loadings of SNF, density, protein, lactose, salts, added water, and conductivity were positive. The second factor weighed heavily on fat, freezing point, and pH. Figure 1b demonstrates the factor scores of the two latent factors. Factor 1, on the horizontal axis, demonstrates the clustering of milk from the GZ farm on the top right side of the scatter plot, while the MT milk samples scattered more on the lower left side. This demonstrates the distinction of the milk quality with respect to proximate values between the two farms. This provides a better picture of what has been observed with the ANOVA discussed above.

“Essential minerals” (Ca, Fe, Zn, Cu, and Se) are those required for the complete life cycle of an organism, the absence or insufficiency of which in the human diet could result in modifications of metabolic functions and some diseases. However, some essential metals become “toxic” when their concentration is high, especially at levels exceeding 40- to 200-fold (Rao, 2005). Two typical toxic metals are Cd and Pb. Several recent investigations show that the presence of toxic metals in foods is observed worldwide (Tunegovà et al., 2016). Studies show that high levels of Cd and Pb in milk are related to external sources and environmental pollution (Sola-Larrañaga and Navarro-Blasco, 2009; Malhat et al., 2012). Öhrvik et al. (2006) reported that developmental problems observed in mice sucklings are due to the dysfunctional effects of high levels of Cd on the mammary glands. The term “heavy metals” poorly describes the nature of metals (Duffus, 2002). In the present study, the term “toxic metals” refers to Ba and Pb, while “essential metals” refer to copper (Cu) and zinc (Zn).

**Table 6:** Milk mineral content in two different farms during the observed period.

Mineral	Farm	Weeks				Farm	P
		1	2	3	4	Average	
Zn, mg L${}^{-1}$	Malta	0.10	0.10	0.10	0.10	0.10	0.000
	Gozo	0.10	0.10	0.00	0.00	0.05	
	Week average	0.1${}^{\text{a}}$	0.1${}^{\text{a}}$	0.05${}^{\text{b}}$	0.05${}^{\text{b}}$		0.000
Cu, mg L${}^{-1}$	Malta	0.5	0.8	0.8	0.7	0.70	0.000
	Gozo	0.73	0.8${}^{\text{b}}$	0.8${}^{\text{b}}$	0.7	0.75	
	Week average	0.61${}^{\text{a}}$	0.8${}^{\text{b}}$	0.8${}^{\text{b}}$	0.7${}^{\text{c}}$		0.000
Pb, mg L${}^{-1}$	Malta	1.00	0.90	1.2	1.1	1.05	0.000
	Gozo	1.26	1.36	1.50	1.66	1.45	
	Week average	1.13${}^{\text{a}}$	1.13${}^{\text{a}}$	1.35${}^{\text{b}}$	1.40${}^{\text{c}}$		0.000
Ba, mg L${}^{-1}$	Malta	1.00	1.00	1.00	1.00	1.00	ns
	Gozo	1.00	1.00	1.00	1.00	1.00	
	Week average	1.00	1.00	1.00	1.00		ns

Ns: not significant. ${}^{a,b,c}$ represent significant differences among weeks at a confidence level of 95 % (p<0.05).

Results on mineral content are presented in Table 6. The GZ farm exhibited higher Pb and Cu content than MT. Lead concentrations in both farms exceed the maximum limit of 0.02 mg kg${}^{-1}$ (EU Regulation 2001/466 for bovine milk). Due to its wide use in industrial processes, lead ranks as the metal with the largest atmospheric diffusion (Soriano et al., 2012). In most studies the concentrations of toxic heavy metals and trace elements were determined in milk obtained from Holstein–Friesian cows (Pilarczyk et al., 2013). It is possible that differences in these metals may be attributed to contamination from milking equipment and recipients that come in contact with the milk.

The mean Cu contents were 0.70 and 0.75 mg L${}^{-1}$ in MT and GZ farms, respectively, during the 4-week observation. The values found agree with results of Meshref et al. (2014), who found Cu values equal to 0.6 mg kg${}^{-1}$. Moreover, Bilandžić et al. (2011) reported Cu concentrations ranging from 0.1 to 0.9 mg L${}^{-1}$, which fall within the normal range in milk. However, a statistical difference between the two farms would not be significant from a health point of view, as long the values fall within the mentioned range. Pilarczyk et al. (2013), in a study on Simmental and Holstein–Friesian cows from the organic farm, found particularly low Cu concentrations amounting to 0.0377 and 0.0453 mg L${}^{-1}$, respectively, indicating the deficiency of this element in animals and thus in the environment and feed. Copper deficiency is a common nutritional problem in ruminants reared in an extensive system, which is not the case with this study.

Concerning Zn concentration, the MT farm showed higher results than GZ, though these were generally lower than those reported in the literature. Reference values of milk concentrations of Zn range from 2 to 6 mg L${}^{-1}$ (Perween et al., 2013; Park et al., 2007; Knowles et al., 2006). However, very low values were recorded, for example, in cow milk in Croatia (0.51±0.16 mg kg${}^{-1}$) (Sikirić et al., 2003). Similarly, McCaughey et al. (2005) found concentrations close to the analytical detection limit. Barium is not considered an essential element to animals or to humans. Its presence in livestock is therefore considered to indicate contamination by extraneous sources (NRC, 2005). For the general population, including children, oral exposure would be the predominant route of exposure (US EPA, 2005). In the present study, Ba levels were identical in both farms. Our results (1 mg L${}^{-1}$) agree with those of Dobrzañski et al. (2005), who assessed the concentration of microelements and trace elements in milk from cows kept in farms located in an industrialized region of the Silesian macro-region, with Ba values ranging 0.19–0.22 mg L${}^{-1}$. In humans, the primary route of exposure to Ba appears to be from the ingestion of food and drinking water as Ba is found in many food groups. Daily dietary intake of barium, especially from milk, flour, potatoes, and nuts, is 0.3–1.8 mg (Głębocka et al., 2014). People can limit exposure to foods and water known to contain barium. However, the amount of barium in foods and drinking water is usually too low to cause health concerns. The WHO (1990) reported several published estimates of dietary intake of Ba by humans; daily dietary intake ranged from 0.3 to 1.77 mg Ba d${}^{-1}$, with wide variations. This is equivalent to 0.004–0.025 mg Ba kg${}^{-1}$ of body weight per day, assuming a 70 kg adult body weight. Barium is also generally present in air as a result of industrial emissions, particularly from the combustion of coal and crude oil, waste incineration, and sometimes from soils.

## 5 Conclusions

In conclusion, the use of hydroponic culture modified the chemical composition of milk with respect to fat content, which is a desirable parameter. Moreover, principal component analysis revealed that with respect to proximate analysis, the quality of milk from cows from the GZ farm was superior to that of cows from the MT farm. The difference between minerals in the two farms may not be easily interpreted as the environment plays an important role, particularly with contaminants in the air, water, and soil.

Further studies are needed to establish the effects of long-term feeding with different types of hydroponic fodder and to investigate the effects of these hydroponic fodders on the productive and reproductive performance of dairy cows. Further studies may be directed towards the development of feeding strategies with respect to the inclusion of hydroponic fodder under different agroclimatic conditions.

## Data Availability

Data are available from the corresponding author upon request.
